# Building a foundation for clinical mass spectrometry and improved patient standard-of-care

**DOI:** 10.1016/j.clinms.2016.10.001

**Published:** 2016-10-26

**Authors:** David Herold, Chris Herold

**Affiliations:** CEO, MSACL, Del Mar, California, United States; Professor of Pathology, University of California, San Diego, United States; President & COO, MSACL, Del Mar, California, United States; Managing Editor, Clinical Mass Spectrometry

Clinical Mass Spectrometry. It’s here. If you have been attending MSACL over the past five years then you have probably been part of the discussion. We thank you for your participation, your conversation, your belief and your willingness to embark on this adventure of establishing an electronic journal that will integrate the field, and facilitate its movement through the next stages of scientific discovery and clinical implementation. While other journals provide some coverage of clinical mass spectrometry research, it is clear that their coverage is limited in scope and detail; they cannot be expected to exert the necessary focus to build the intensity and momentum needed to fully leverage the potential of our field. As the clinical application of mass spectrometry matures, and increasingly impinges on the status quo of laboratory practice, the community will require a dedicated and uncompromised champion, not only for bridging the technology to other scientists and the public, but to align discussions and act as a communal touchstone. Submissions to Clinical Mass Spectrometry will not only strengthen the field, they will increase the relevance and legitimacy of your research. Historically, few have had the chance to contribute tangible benefit to the evolution of analytical methods to improve patient outcome and cost-containment. You can now share your intellect with a dedicated community to build a unified vision. We look forward to working with you to capitalize on this unique opportunity to improve patients’ standard-of-care.



Sincerely,

